# New site at Olduvai Gorge (AGS, Bed I, 1.84 Mya) widens the range of locations where hominins engaged in butchery

**DOI:** 10.1038/s41598-022-14031-1

**Published:** 2022-06-13

**Authors:** Lavinia M. Stancampiano, Ainara Sistiaga, David Uribelarrea del Val, Julia Aramendi, Enrique Baquedano, Audax Mabulla, Manuel Domínguez-Rodrigo, Clayton R. Magill

**Affiliations:** 1 Lyell Centre at Heriot Watt University, Edinburgh, UK; 2 University of Copenhagen, Copenhagen, Denmark; 3 Massachusetts Institute of Technology, Cambridge, USA; 4 Institute of Evolution in Africa (IDEA), University of Alcalá, Madrid, Spain; 5 Complutense University, Madrid, Spain; 6 University of Salamanca, Ávila, Spain; 7 Regional Archaeological Museum of the Community of Madrid, Madrid, Spain; 8 University of Dar es Salaam, Dar es Salaam, Tanzania; 9 Department of Anthropology, Rice University, Houston, TX 77005-1827 USA

**Keywords:** Lipids, Anthropology, Archaeology, Ecology, Environmental chemistry

## Abstract

Outstanding questions about human evolution include systematic connections between critical landscape resources—such as water and food—and how these shaped the competitive and biodiverse environment(s) that our ancestors inhabited. Here, we report fossil *n*-alkyl lipid biomarkers and their associated δ^13^C values across a newly discovered Olduvai Gorge site (AGS) dated to 1.84 million years ago, enabling a multiproxy analysis of the distributions of critical local landscape resources across an explicit locus of hominin activity. Our results reveal that AGS was a seasonally waterlogged, largely unvegetated lakeside site situated near an ephemeral freshwater river surrounded by arid-adapted C4 grasses. The sparse vegetation at AGS contrasts with reconstructed (micro)habitats at the other anthropogenic sites at Olduvai Gorge, suggesting that central-provisioning places depended more heavily on water access than vegetation viz. woody plants as is often observed for modern hunter-gatherers. As hominins at AGS performed similar butchering activities as at other Bed I sites, our results suggest they did not need the shelter of trees and thus occupied a competitive position within the predatory guild.

## Introduction

Olduvai Gorge contains one of the most important records of hominin remains, archaeological deposits, and fossil large-mammals in the world^1–3^. Over the last half century, myriad hominin environments at Olduvai have been interpreted as grasslands to open woodlands with a mosaic of grassy groundcover and thickets^4–6^. Yet, the spatial distribution of landscape resources and early hominin interactions within their immediate surroundings (microhabitat) remains unresolved.

Previous work at Olduvai focussing on Bed I times (2.0–1.8 Mya^7^) suggests that the vegetation and (hydro)climatic changes documented therein correlate with orbitally (precession) driven cycles superposed on a wider aridification trend^8,9^ that continues into the modern day^10^. Cyclical 19–23 kyr insolation cycles triggered hydroclimate variations in the catchment basin, which in turn drove ecological transitions viz. closed forests during wetter periods to arid C4 graminoid-dominated environments^4–6^ in-step with transgression–regression phases of the saline, alkaline nearby paleolake Olduvai^11,12^. Rare species-specific plant remains, including phytoliths and large macrofossils, indicate vegetation was dominated by trees and monocotyledonous vegetation, including palms, sedges and *Typha* cattails^13^ among patchy (paleo)wetlands^14^. The distributions of locally-occurring plants had direct implications on hominin behaviour at Olduvai Gorge and yet remains undiscerned because of limited availability of high-resolution, contiguous samples across discrete archaeological horizons with in situ artifacts and flora indicators^15^.

**Figure 1:**
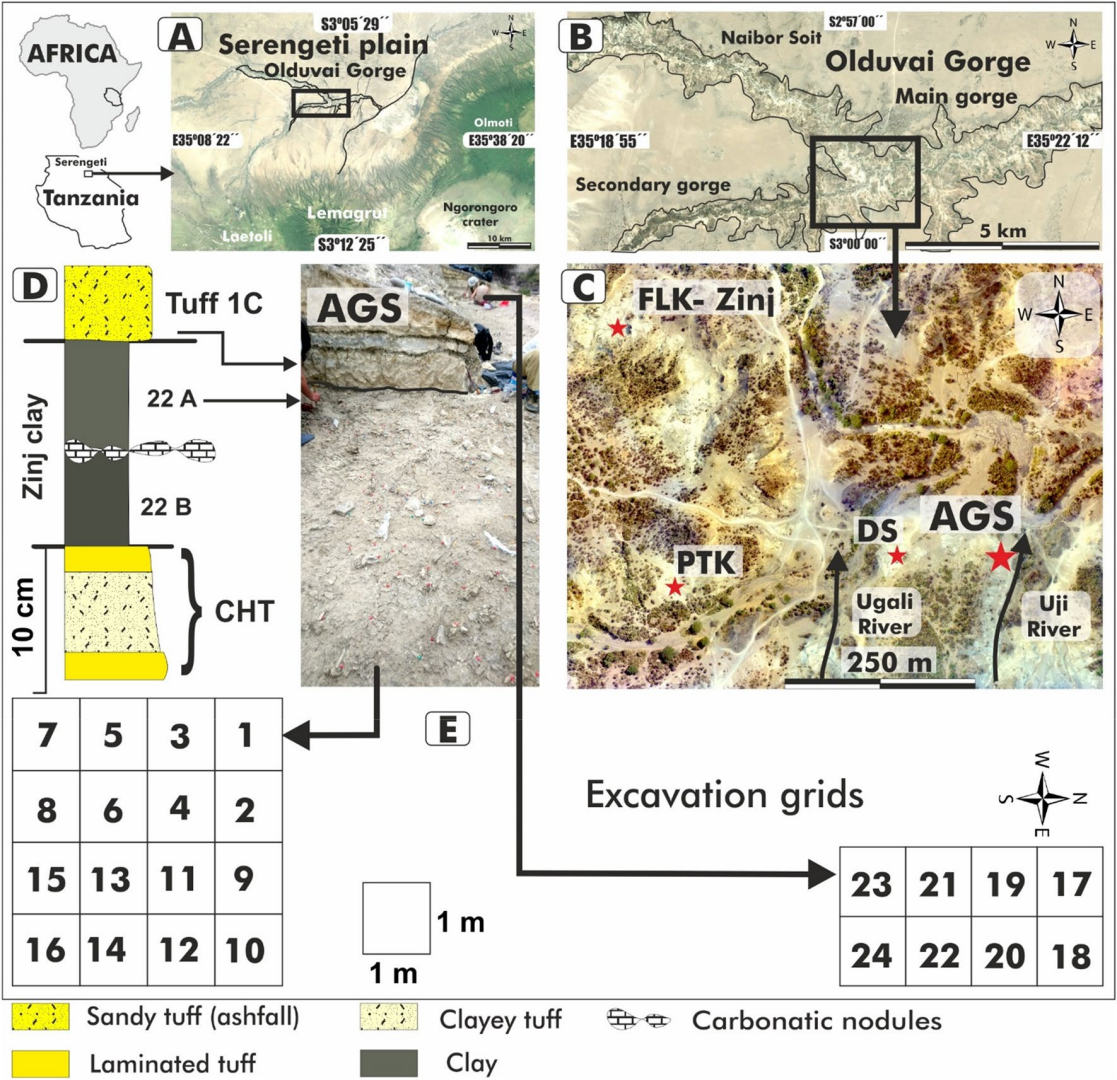
Study area and sample locations at Olduvai Gorge, Tanzania. (**A**,**B**) Contemporary geographical location of Olduvai Gorge. (**C**) Detailed map of the study area. Red stars indicate archeological sites contemporaneous with the Zinj Paleolandscape. Black arrow lines represent the flow direction of the paleo-rivers Ugali and Uji. (**D**) Stratigraphic section at AGS level 22A, which lies between Chapatti Tuff (CHT) and Tuff 1C. (**E**) Archeological excavation grid at AGS for spatial biomarker reconstructions, including photo of the excavation surface. This figure was made using ArcGis 10.6.1 https://www.esri.com/fr-fr/arcgis/products/arcgis-desktop/resources.

Here, we focus on a newly discovered archaeological site at Olduvai, called AGS (Alberto Gómez Site) (Fig. 1B). The uppermost horizon of AGS lies in Bed I below Tuff IC, dated to 1.832 ± 0.003 Ma^7^, and is isochronous with the clay stratum level 22A (Fig. 1D) that forms much of the coeval ‘Zinj Paleolandscape’^16^. The Zinj Paleolandscape—which includes FLK Zinj-DS-PTK-AMK and the new AGS site (Fig. 1)—offers an exceptional opportunity to study very-well preserved assemblages over an extensive contiguous area^16^.

We report the results for a combined lipid biomarker and isotope reconstruction of 24 m^2^ of (hydro)ecological landscape at AGS that, together with existing piecemeal landscape reconstructions around the Zinj Paleolandscape, provides new and high-resolution insight into emerging patterns of hominin local land-use and behavioural dynamics amid human evolution in a changing global climate.

## Results and discussion

### Plant biomarker distributions

Plant lipid biomarkers are widely used to reconstruct vegetation and (hydro)climate in ancient environments, and previous studies at Olduvai^15,17^ have used biomarkers in spatiotemporal landscape reconstructions, although not at the resolution of this study (i.e., < 1 m^2^). Here, we also expand the molecular scope of these earlier studies with an explicit focus on complementary plant biomarkers viz. *n*-alkanes, *n-*alkanols, and *n*-alkanoic (fatty) acids. Combined, these *n*-alkyl lipids offer unique insights into spatial landscape resource distributions well beyond that from any particular individual biomarker class^18^.

### Alkanes and alkanols

**Figure 2:**
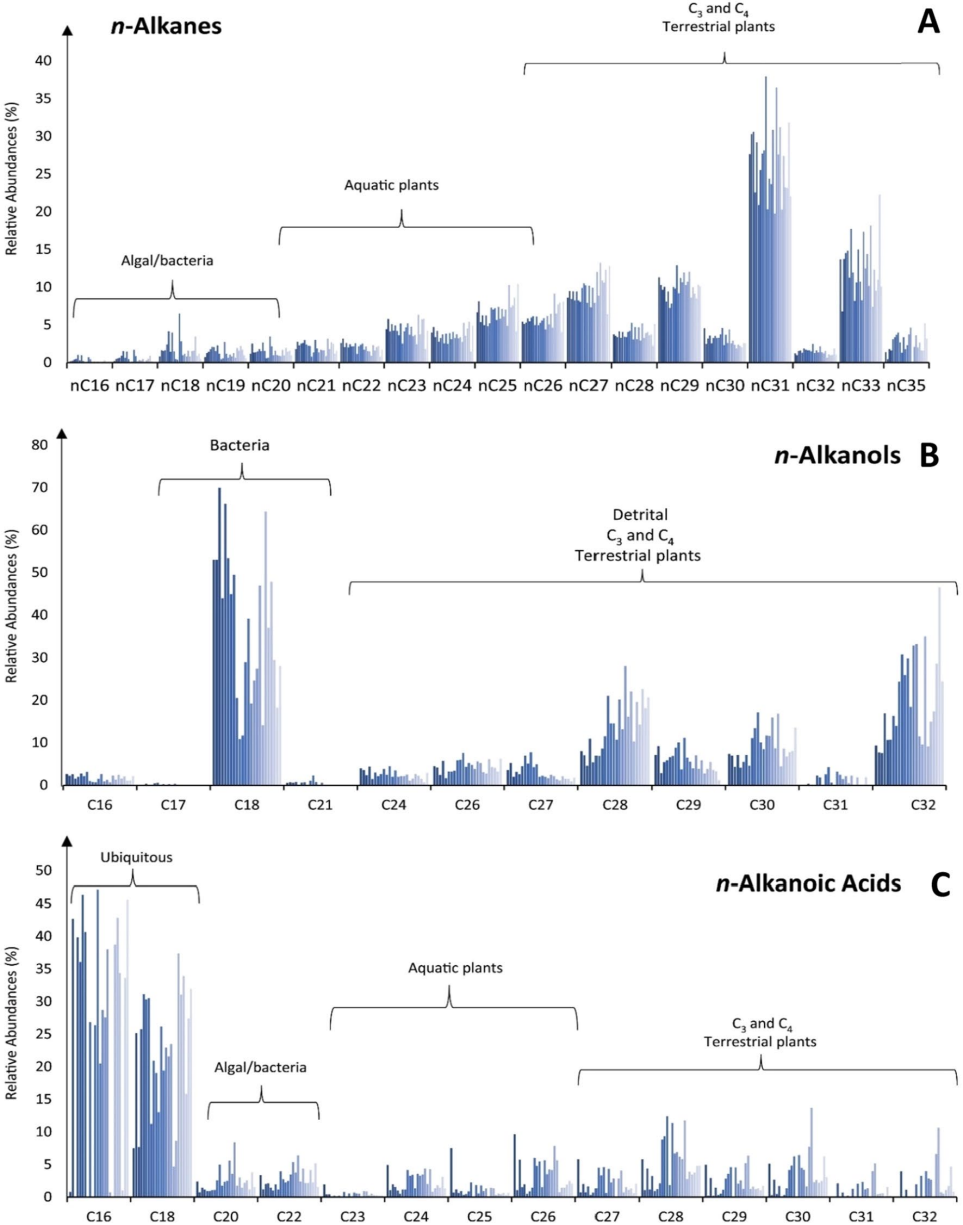
Lipid distributions and the relative abundances of key polarity fractions identified through GC–MS analyses. (**A**) n-Alkanes; (**B**) n-Alkanols; (**C**) n-Alkanoic acids. Diagnostic source associations are also shown^19,20,24–26,35,65^.

**Figure 3:**
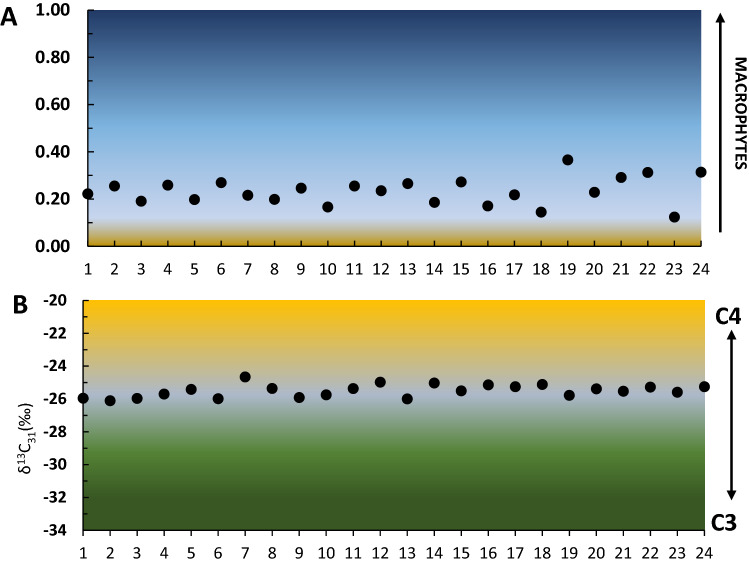
Molecular and isotopic indicators used for vegetation reconstruction at AGS. (**A**) Macrophyte-derived lipids (nC_23_ + nC_25_) relative to macrophytic and terrestrial lipids (nC_23_ + nC_25_ + nC_29_ + nC_31_). Values of < 0.4 indicate no macrophytes; values of 0.4–1 indicate emergent macrophytes; values > 1 indicate floating macrophytes^19^. Colouring gradient denotes relative macrophyte input. (**B**) Plant-lipid δ^13^C_31_, in which higher values indicate more C4 vegetation input.

All samples at AGS yielded substantial amounts of homologous *n*-alkanes spread between *n*C_16_ and *n*C_35_ (Fig. 2A) with a distinct dominance of long-chain, odd-numbered homologues (ACL = 29.5) (Fig. 6D), indicative of both aquatic and higher plant inputs^15,19,20^. With this in mind, P_aq_ values —a ratio of the aquatic-derived lipids relative to aquatic and terrestrial lipids in toto^19^ (Fig. 3A)—indicate the proportional dominance of floating/submerged (i.e., aquatic) macrophytes as compared to emergent macrophytes and terrestrial plants. Samples at AGS feature consistent P_aq_ values of between about 0.1–0.4 (Fig. 3A) indicative of high input from floating and emergent macrophytes such as cattails (*Typha* sp.). P_alg_ values, a ratio of the algal lipids relative to algal plus terrestrial lipids^20^, of < 0.1 further indicate nominal lipid input from algae at AGS (Fig. 6C) that is inconsistent with swamp or salt-marsh habitats, which are rich in edaphic algal biomass^6,21^. Together with intermediate values of the *n*C_33_/*n*C_31_ ratio of 0.2–0.7 (average of ~ 0.6), in which higher values suggest increasing grass cover^22,23^, we interpret AGS to have been covered in mostly graminoids.

Analysis of intermediate polar (i.e., functionalized) lipid fractions illuminate crucial details about the paleoenvironment at AGS that biomarker *n*-alkanes alone cannot. Major *n*-alcohols in our sample extracts include mid- (C_14_ to C_22_) and long-chained (C_24_ to C_32_) with a strong predominance of even-numbered homologues (Fig. 2B). Most samples’ *n*-alkanol distributions are dominated by *n*C_18_OH that is characteristic of bacteria and freshwater algae inputs^24–26^. However, several distributions are rather dominated by phytoplankton-derived *n*C_24_OH^24,25^ or commonly plant-derived *n*C_28_OH^25^, further clarifying the complex mixture of both aquatic and terrestrial lipid inputs. Common occurrences of *n*C_26_OH, typical of freshwater microalgae (*Eustigmatophyceae*)^24,26^, further indicates regular inundation with flowing potable water^18^ at AGS.

Previous studies suggest the ratio of *n*-hexacosanol (*n*C_26_OH) to *n*-nonacosane (*n*C_29_)—called the alcohol preservation index (API)—reflects changes in oxygenation at the sediment depositional interface^27,28^, wherein smaller values indicate more oxic conditions. At AGS, API values range from 0.2 to 0.4 (Fig. 6A) that fall between average values of hypoxic (API > 0.4) and oxic (API < 0.2) conditions^27^. Based on these values, we interpret API values at AGS to indicate seasonal flooding pulses across a riparian wetland^29^ that drove cyclic organic matter oxidation via oxygen exposure at the sediment–water interface after inundation^30^. Moreover, all samples from AGS lack detectable concentrations of the isoprenoidal lipids pristane and phytane. Because pristane/phytane are dominantly produced from chlorophyll degradation^31,32^, and (photo)degradation rates are rapid in most fluviolacustrine systems^33^, lacking pristane/phytane at AGS is consistent with (sub)seasonal redox oscillations, aerobic conditions and high-light intensities characteristic of barren medial floodplain channel deposits with little standing plant biomass^34^.

### n-Alkanoic acids

The polar fractions of AGS lipids show an archetypal bimodal distribution of saturated mid- and long-chain *n*-alkanoic acids (i.e., *n-*docosanoic acid [*n*C_22:0_] to *n*C_26:0_ and *n*C_28:0_ to *n*C_32:0_, respectively) (Fig. 2C) with an even-over-odd predominance that is indicative of mixed aquatic and terrestrial higher vegetation inputs, respectively^35^. Even so, shorter-chained homologues viz. *n*C_16:0_ and *n*C_18:0_ dominate among polar moieties at AGS. These shorter-chain acids are ubiquitous among plants, animals and fungi, but are especially prominent in algae and bacteria^21^. Therefore, we used the so-called terrigenous to aquatic ratio (TAR^FA^)—the ratio of long-chain versus shorter-chain alkanoic acids^35^—for determining proportional lipid input from disparate organic matter sources. Low calculated TAR^FA^ values of 0.05 (Fig. 6F) at AGS indicate aquatic or algal lipids dominated biomass input^35^ despite its shoreside setting. The diagnostic occurrence of mono- and polyunsaturated *n*C_22_ alkanoic acids suggest there was a major input from microbes and freshwater phytoplankton^26^, with further supports our interpretation of AGS as a riverside wetland that experienced frequent flooding.

### Stable carbon isotope signatures among plant biomarkers

#### Alkane δ^13^C values

**Figure 4:**
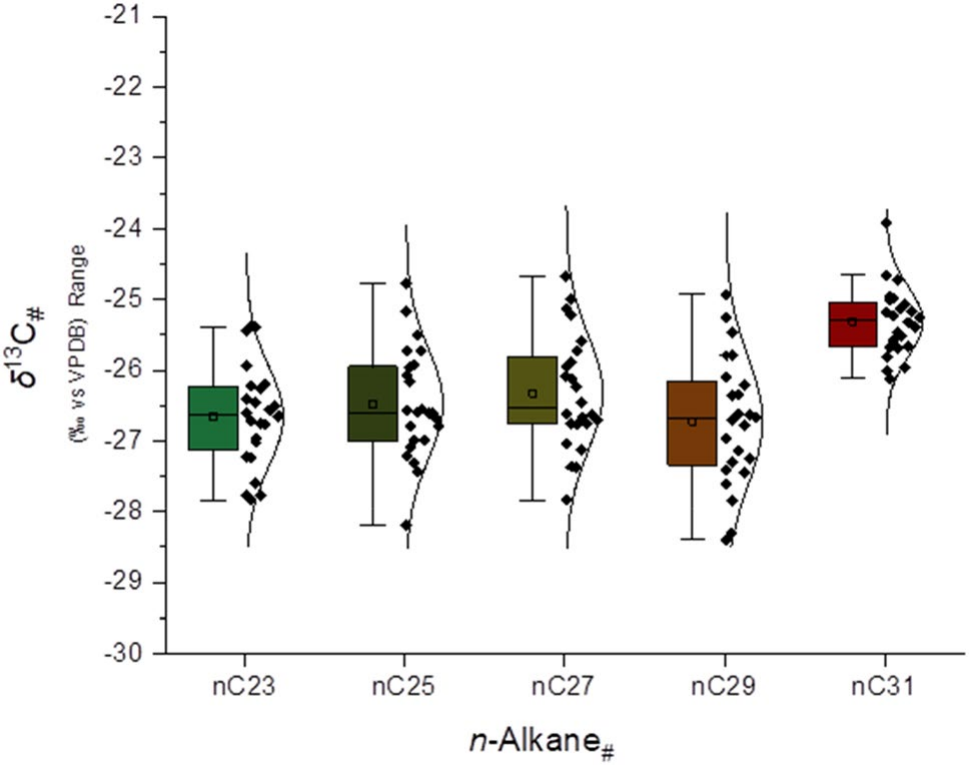
Compiled leaf-wax n-alkane signatures of 24 sediments samples excavated from at AGS. Stable carbon isotopic values of C_23–C31_ n-alkanes (δ^13^C_#_) are shown as median values with their median absolute deviation (± MAD) and ANOVA test distributions. Boxplots depict distributional differences between individual homologue δ^13^C_#_ values.

Previous studies report fossil and tenable molecular evidence of lacustrine vegetation viz. aquatic macrophytes in Bed I sediments at Olduvai^15^, indicating parallelism with wetlands in contemporary East Africa^19^. Molecular isotopes evidence at AGS features similar support through the stable carbon isotopic signatures of *n*-hentriacontane (δ^13^C_31_) that have values of − 25.0 ± 1.1‰ (Figs. 4 and 6H), which we interpret to indicate a complex mixture of C3 and C4 plant inputs^36^. Macrophytes with aquatic (e.g., *Hydrilla*), floating (e.g., *Potamogeton*), and emergent (e.g., *Cyperus* and *Typha*) habits are common in recent East African wetlands^19^, and have similar δ^13^C values as we observe at AGS^19,37,38^. However, macrophytic carbon sources are often influenced by partial incorporation of dissolved HCO_3_^−^ (which causes increased, C4-like δ^13^C values) in aquatic environments, and likewise are affected by physiological differences among plant growth forms and functional types^19,39,40^ that lead to interpretational difficulties of δ^13^C_31_ values vis-à-vis differentiating plants with bicarbonate uptake mechanisms versus cooccurring graminoids with C4 photosynthetic pathways.

To determine source and input differences among plant-lipid biosignatures, we applied basic analysis of variance (ANOVA) to our measured δ^13^C values for odd-numbered homologues (i.e., *n*C_23_, *n*C_25_, *n*C_27_, *n*C_29_, *n*C_31_) in sediments at AGS. In this context, the mid-chain *n*-alkanes (*n*C_23_ and *n*C_25_) (Fig. 6G) are derived from submerged/floating macrophytes, and longer-chain *n*-alkanes (> *n*C_25_) are dominated by plants with terrestrial habitats^39^. Resultant δ^13^C ANOVA data demonstrate a statistical difference (*p*-value < 0.01) between δ^13^C_31_ as compared to contemporaneous mid-chain and longer-chain *n*-alkanes. Our results suggest that *n*C_31_ had a discrete source with more ^13^C-enriched lipids—such as arid-adapted C4 graminoids^41,42^—as compared to other *n*-alkanes. In contrast, the other mid- and longer-chained homologues do not show statistical differences in average isotopic composition. With this in mind, we suggest that the algae and submerged/floating aquatic plants at AGS incorporated higher amounts of dissolved HCO_3_^−^ as a source of carbon, resulting in heavier δ^13^C values in mid-chain *n*-alkanes^40^. Complementary proxies viz. *n*-alkane ratios (Figs. 3A,B, 6A–E) further suggest that AGS functioned like a seasonal-to-permanent freshwater-fed floodplain or river margin dominated by graminoids and C3-like macrophytes with sparse woody plants and limited C4 grasses. This is in accordance with the geological and sedimentological description that suggested a playa lake margin with small rivers with low-energy^11,16^.

#### Alkanoic acid δ^13^C values

**Figure 5:**
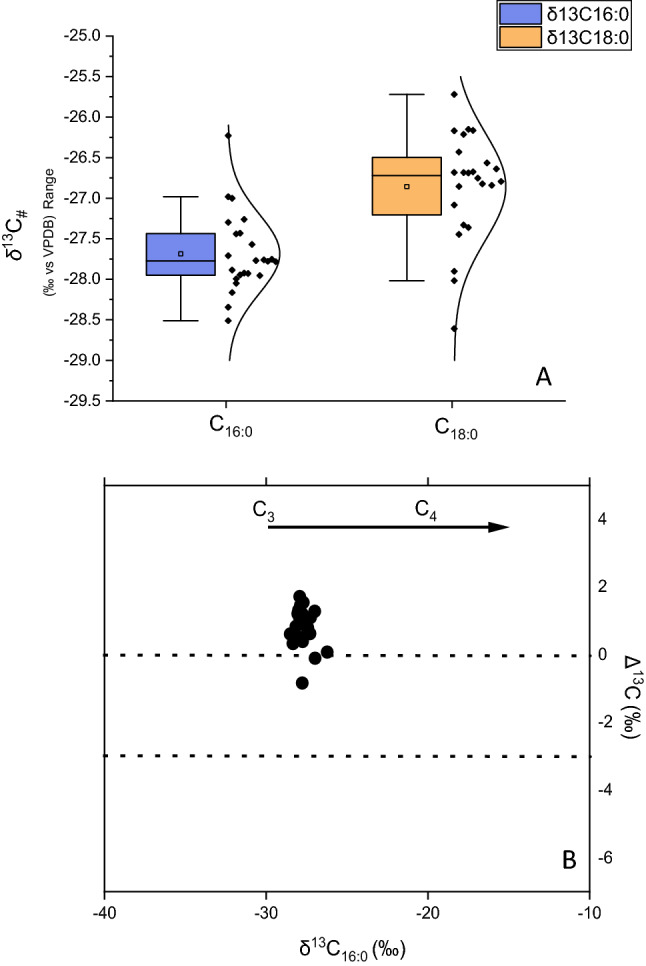
Stable carbon isotope composition (δ^13^C values) of individual fatty acids in sediments excavated from at AGS. (**A**) δ^13^C_16:0_ and δ^13^C_18:0_ values are shown as medians with their median absolute deviation (± MAD) and ANOVA test distributions. Boxplots depict distributional differences between individual homologue δ^13^C_#_ values. (**B**) Qualitative isotopic source allocation of dominant fatty acids extracted from sediments at AGS. Graphical framework modified from Evershed and colleagues^43–45^.

The ubiquity of *n*C_16:0_ and *n*C_18:0_ makes their δ^13^C values useful to differentiate organic matter sources^24^. Individual δ^13^C_16:0_ and δ^13^C_18:0_ values range from − 26.2 to − 28.3‰ with a ~ 2‰ offset that indicates respective organic matter sources had a dominantly C3 photosynthetic pathway^43^ (Fig. 5A). Further, individual δ^13^C_16:0_ and δ^13^C_18:0_ values show similar δ^13^C values as compared to mid-chain (*n*C_23_ and *n*C_25_) and longer-chain (*n*C_27_ and *n*C_29_) alkanes, suggesting both compound classes share similar organic matter sources. The crossplot of δ^13^C_16:0_ against the offset between δ^13^C_16:0_ and δ^13^C_18:0_ (Δ^13^C_18:0–16:0_) demonstrates additional differences in *n*-alkanoic acid inputs derived from C3 and C4 sources^43–45^ (Fig. 5B). Considered together, molecular isotopic values suggest that AGS was a microhabitat defined by a mixture of C3-graminoids with extensive aquatic plant inputs.

### The Zinj Paleolandscape and hominin evolution

At least three hominin taxa—including *Homo* cf. *ergaster*, *Paranthropus boisei* and *Homo habilis*^11,46,47^—have been recovered from the Zinj Paleolandscape. Across this contiguous archaeological horizon, artifacts and fossil bones are embedded within a distinctive silty-clay layer covered by airfall Tuff IC^11^, which suggests that the remains were captured in situ under volcanic ash fallout. Rapid burial fostered the exceptional preservation of fine-sediment features (e.g., root ichnofossils)^16^, macrofossils and biomarkers across its surface^11,16^. Sedimentological features at AGS further reveal that this site was situated at littoral–supralittoral interface, where alluvial fans and floodwater river runoff interposed the mudflat littoral zone^16^. Altogether, such multidisciplinary proxies reveal that Olduvai, and the Zinj Paleolandscape especially, presented hominins with a variegated landscape of heterogeneous resource distributions, and thus impacted hominin diets and behaviour.

Diet is considered to serve as a major selective force amid hominin encephalization^48^, and the intake of ‘essential’ lipids (e.g., polyunsaturated fatty acids), which are uniquely prevalent in wetland flora^49,50^, is critical to cerebral development in modern infant humans^51^. Aquatic macrophytes proliferate in wetlands today in East Africa^19,52^ and likely provided hominins with high-quality provision viz. rootstocks and leaves all year long^53,54^. Contemporary pan-African macrophytes rarely use dedicated C4 photosynthesis; but, these (semi)aquatic plants can incorporate bicarbonate when in alkaline waters and, in doing so, create C4-like δ^13^C isotopic signature^40^. Mid-chain *n*-alkanes from AGS reveal ^13^C-enriched biomass (Fig. 4), and that their source vegetation (i.e., macrophytes) can account for high δ^13^C signatures in hominin diets, as reflected through bioapatite isotopes and dentition analyses^55–58^.

**Figure 6:**
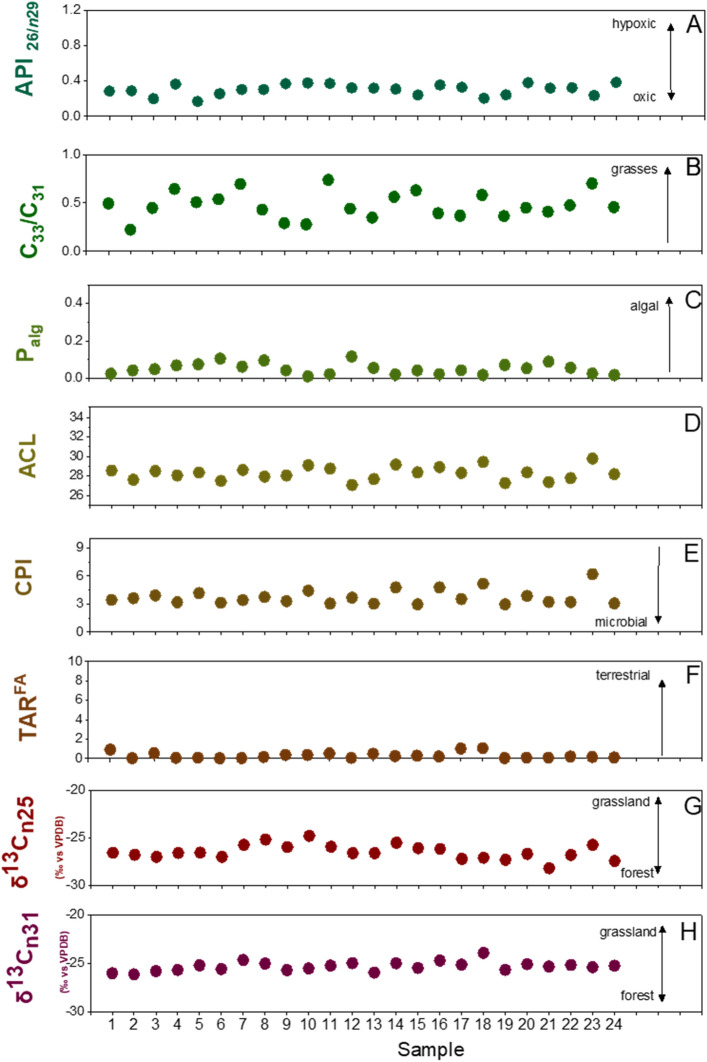
Key proxies measured for sediments excavated from at AGS. (**A**) API: alcohol preservation index uses only n-hexacosanol and n-nonacosane. Higher values indicate more hypoxic conditions^27,28^. (**B**) C_33_/C_31_: ratio of nC_33_ to nC_31_. Higher values indicate more grasses^22,23^. (**C**) P_alg_: ratio of algal lipids (nC_17_ + nC_19_) relative to algal and terrestrial plant lipids (nC_17_ + nC_19_ + nC_29_ + nC_31_). Higher values indicate more algal input^20^. (**D**) ACL = average chain length of individual n-alkane abundances^20^. (**E**) CPI = [∑odd(C_21–33_) + ∑odd(C_23–35_)]/(2∑even C_22–34_): carbon preference index, which is indicative of the abundance of odd over even carbon chain lengths. In general, lower CPIs indicate microbial inputs or maturation of the sample^20^. (**F**) TAR^FA^: terrigenous to aquatic n-alkanoic acids ratio reflecting the importance of terrigenous as compared to aquatic sources (C_24_ + C_26_ + C_28_)/(C_14_ + C_16_ + C_18_). Higher values indicate more terrestrial input^35^. (**G**,**H**) Plant-lipid δ^13^C values for n-alkane C_25_ and C_31_. Higher values indicate more C4 plant dominated environment^40^.

Our molecular and isotope data (Figs. 3A, Fig. 6A,C) suggest there was a intermittently-waterlogged floodplain environment at AGS, which is consistent with earlier mineralogical descriptions at the vicinal DS site (Fig. 1C)^59^. We suggest there was a notably perennial large stream or river near AGS that flowed from south-to-north^16^ into nearby paleolake Olduvai, and brought with it the allochthonous plant lipid from nearby grasslands and forest patches^13,15^. Given overall low relief in the immediate area around AGS, which likely produced lower-energy paludal flow, entrainment of allochthonous materials most likely peaked through seasonal flooding pulses^60^. In any case, associated freshwater fluvial input must have attracted both hominins and large carnivores alike to AGS.

Biomarkers at AGS build upon earlier reconstructions at Olduvai^6,17,22^ and the Zinj Paleolandscape^15^ that imply plants and freshwater distribution exerted direct influence on hominin behaviour. However, in contrast to earlier reconstructions among penecontemporaneous sites at Olduvai Gorge, our data at AGS indicates hominins took strategic advantage of low- or unvegetated locations in addition to dense woodland thickets, low-visibility papyrus stands, and tall grassland^13,61^. AGS itself likely harboured occasional large woody plants such as *Hyphaene* palms^13^, but the abundance of this vegetation would have been nominal^62^.

The occurrence of discrete plants on otherwise unvegetated fluviolacustrine surfaces might be one explanation for differing paleoecologic reconstructions with biomarkers, which integrate immediate and upstream organic matter inputs, as compared to, e.g., phytoliths, which capture only productive in situ vegetation and do not transport downstream^63^. Regardless, vicinal (over)bank deposits and levee ridges would be dominated by generally groundcover vegetation^29^ such as macrophytes, pteridophytes (e.g., ferns) and low woody plants, meaning that hominins at AGS would have an unencumbered view of the surrounding landscape^64^, including precious refuge about 400 m away at FLK Zinj itself^13,15,16^ (Fig. 1C). Biomarkers across the Zinj Paleolandscape^15^ and from the AGS site show distributions of plants that suggests critical resources had a direct implication on hominin behaviour at Olduvai Gorge.

**Figure 7:**
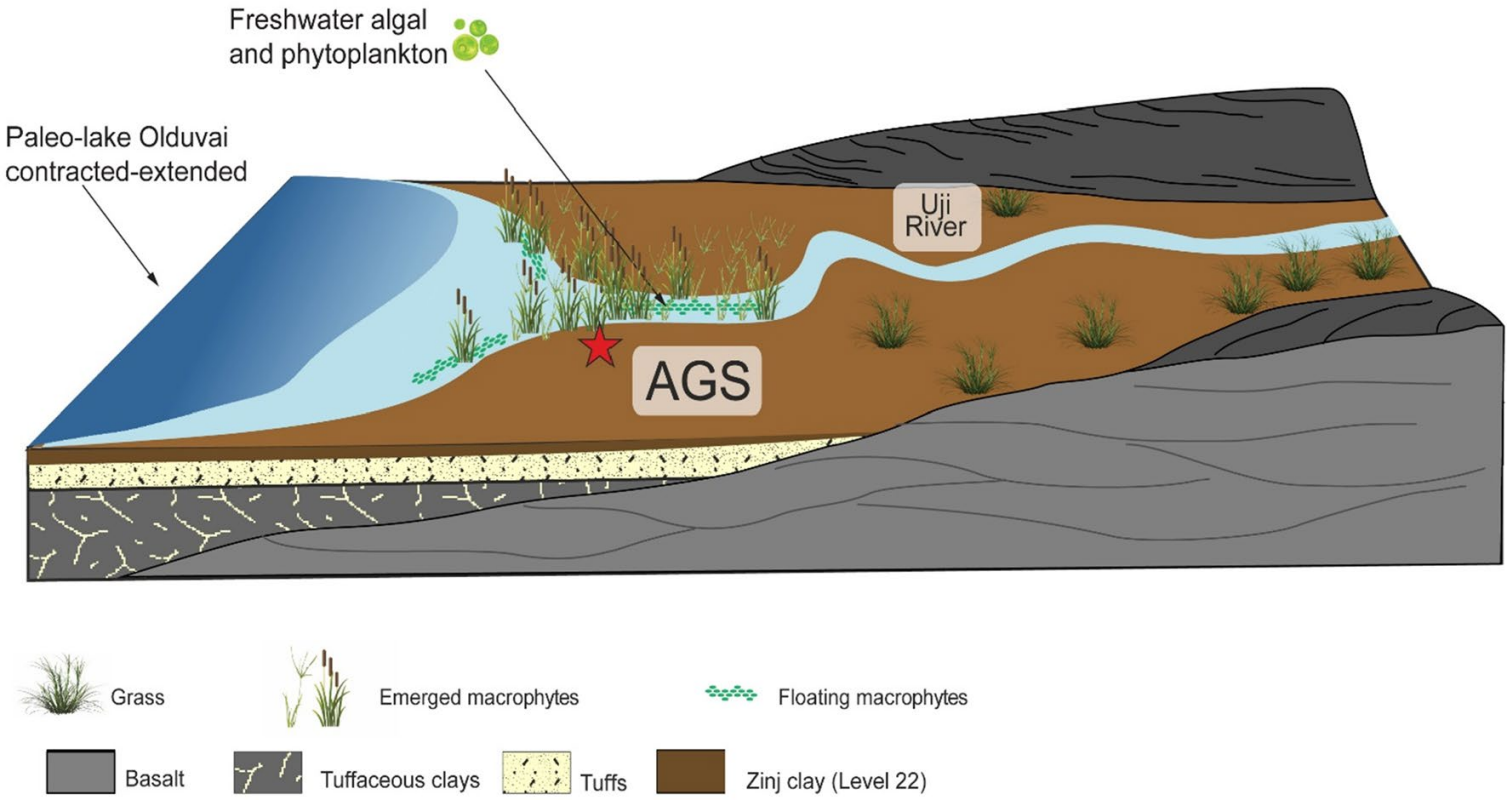
Graphical three-dimensional geomorphological reconstruction of the syndepositional Zinj level 22A horizon at AGS between two lava tongues and Uji River. AGS was located near a river and the main vegetation inputs were from emerged macrophytes, floating macrophytes, freshwater algal and phytoplankton. This figure was made using Adobe Creative Suite 6 for Windows https://www.adobe.com.

The AGS archaeological site contains one of the highest densities of faunal remains on the Zinj Paleolandscape after DS, which to date is the biggest window into an Early Pleistocene anthropogenic site^65^. This suggests that the area at AGS must have been occupied for repeated instances of large carcass consumption^65^. To boot, AGS was dominated by (semi)aquatic plants, minimal trees or shrubs, although in all likelihood hominins were occupying the site especially for its vicinity to flowing water. In contrast, FLK NN (500 m west from AGS) contained limited faunal remains and lithics within an environment that featured floating and submerged aquatic plants^15^ located nearby freshwater carbonates (tufa^8^). If AGS was not a terrestrial plant-dominated habitat, because of its barren vegetation, and hominin and carnivore visibility were similar, as in open grasslands, this posits the question of how hominins efficiently avoided carnivore hazards for the prolonged occupation(s) inferred from the intensity of ungulate carcass butchery documented at the site (Fig. 7). This is mostly notable, especially given the paucity of taphonomic evidence signalling carnivore modification of carcass remains. The evidence is suggestive of hominins having carved a competitive niche against other predators by efficiently fending off their hazard.

By comparing our results at AGS to previously published data about biomarker distributions amid the Zinj Paleolandscape^15^, we suggest that AGS functioned as a seasonally waterlogged, low-vegetation environment characterised by dense accumulations of butchery-process debris within a wider mosaic environment that harboured both open^5,15^ and dense, closed ecotones, such as at DS [57]. FLK Zinj, DS and PTK are the other three anthropogenic sites occupying the same thin clay stratum as AGS on the Zinj Paleolandscape [63], [107]. These three sites were created in a wooded to forested alluvial environment [57], [108]. The close spatial association between stone tools and fossil bones indicates a functional relationship resulting from butchery. This is further supported by the abundant cut-marked and percussed bones retrieved in the archaeofaunal assemblage, which is currently under analysis. Given that in the other anthropogenic sites, with areas sampled similar to the size of the area sampled at AGS, the paleobotanical signal is of closed vegetation, we assume that the area sampled at AGS is equally representative of the main vegetational characteristics of the setting. In this case, the signal retrieved, in contrast with the other anthropogenic middle Bed I sites is of a fairly open environment. The butchery patterns documented in those sites are identical to those observed at AGS (work in progress), with a similar number of animals and taxa represented and a similar single-round cluster site structure. This latter feature is clearly showing a carcass butchery and consumption pattern and a distinctive use of the space that contains crucial socio-reproductive information [109]. To conclude, we suggest the spatial landscape ecology patterns defined both by macro- and molecular fossils reveal hominin engagement in social transport of large resources, such as bringing animal carcasses and freshwater-sourced food to AGS from the surrounding grasslands and lakeside environments.

## Conclusions

Our multi-proxy geochemical interpretations of the new site called AGS at Olduvai Gorge ca. 1.84 Ma reveal that it was a sparsely vegetated, high-visibility paludal location on a riverbank within a wider mosaic environment defined by the Zinj Paleolandscape. Standing plant biomass at AGS was dominated by aquatic vegetation viz. macrophytes and C4 graminoids, consistent with its riparian and lake margin setting. Comparison with literature data for coeval localities at Olduvai Gorge suggest that this ancient landscape was rich in ecotones with abrupt transitions between disparate vegetation communities, as is often observed today in East African wetlands. Given the markedly patchy paleoenvironment that defined the vicinal Zinj Paleolandscape and the unique (sub)seasonal floodplain at AGS, we conclude early hominins at Olduvai Gorge selected locations for cooperative resource processing—such as animal butchery—as related to water resources rather than refuge (i.e., closed thickets). This conclusion diversifies the environments in which vertically discrete anthropogenic sites occur and furthermore insinuates that hominins felt equally at ease in such environments. Considered together, new and old data at Olduvai reveal that hominins had reached an adaptive carnivore status by 1.84 Ma that enabled them to cope with terrestrial predation risks and fend off other carnivore competitors.

## Materials and methods

### Sampling

Samples were collected from sediment 0–2 cm below Tuff IC that is isochronous with Zinj level 22A, in a silty-clay layer where bones and lithic tools occurred in situ (Fig. 1C). Representative sediment samples (~ 50 g; *n* = 24) from individual 1 m^2^ gridding quadrats (Fig. 1D) were collected between fossil remains and stone tools with a clean aluminium spoon.

### Biomarker extraction and isolation

Sediments were freeze-dried and powdered with a clean agate mortar and pestle. Lipids were extracted from sediments via an accelerated solvent extractor (Dionex ASE 350 system) with dichloromethane (DCM) and methanol (MeOH) (9:1 vol/vol) in 3 cycles at 100 °C (10.3 MPa) with static time of 5 min. Resultant total lipid extracts (TLE) were dried beneath a gentle stream of nitrogen, and then derivatized through acid methanolysis (0.5 M HCl in MeOH diluted with ultrapure water) before subsequent liquid–liquid isolation into hexane:DCM (4:1 vol/vol)^17^. Derivatized TLEs were concentrated and chromatographically partitioned into three fractions using deactivated silica gel (2% H_2_0 by weight) by elution with hexane (F1), hexane:DCM (1:1 [F2]) and DCM:MeOH (4:1 [F3]). Polar (F3) fractions were silylated using N,O-bis(trimethylsilyl) trifluoroacetamide (BSTFA) for detection as trimethylsilyl derivatives.

### Molecular analysis

Lipid biomarkers were characterized by gas chromatography-mass spectrometry (GC–MS; Thermo Scientific TRACE 1310 [GC] with coupled ISQ LT [MS]) by splitless injection of 1 μL aliquots of individual lipid fractions onto a 60-m VF1 fused-silica column (0.25 mm × 0.25 μm). The GC oven was programmed to: 60 °C injection and hold for 2 min, ramp at 10 °C min to 150 °C, ramp at 4 °C min to 300 °C followed by isothermal hold of 20 min. The transfer line and source were set at 320 °C and 270 °C, respectively. Procedural blanks were run to monitor contamination and background interferences. Compound identifications were made via comparison with authentic standards in conjunction with the NIST20 electron ionization spectral library.

### Isotopic characterization

Target biomarkers were analysed for their isotopic signatures by gas chromatography combustion isotope-ratio monitoring mass spectrometry (GC-C-irMS) using a TRACE 1310 interfaced to a GC Isolink II connected to a Conflo IV and Delta V Plus. The GC oven was programmed to: 60 °C injection and hold for 2 min, ramp at 4 °C min to 320 °C followed by isothermal hold of 10 min. An aliquot of 1 μL was injected in splitless mode onto a 30-m DB5ms column (0.25 mm × 0.25 μm) before combustion over copper, nickel, and platinum wire with oxygen and helium at 1000 °C. Isotopic values were normalized and corrected using a mixture of *n*-alkanes (*n*C_16_ to *n*C_30_) of known isotopic composition (Mixture B4 [Schimmelmann Standards]) or a mixture of fatty acid methyl esters (FAMEs [C_14:0_, C_16:0_, C_18:0_, C_20:0_]) of known composition (Mixture F8-3 [Schimmelmann Standards]). Standard deviation of the calibration standards is 0.2‰ (*n*-alkanes, *n* = 1152), to 0.3‰ (FAMEs, *n* = 288). Isotopic corrections for carbon added through esterification (0.5 M HCl in methanol) were made via δ^13^C for esterified benzene-1,2-dicarboxylic acid (phthalic acid [Schimmelmann Standards]) that then was used to correct for isotopic mass-balance of derivative carbon.

## Supplementary Information

Supplementary Information.

## Data Availability

All data generated or analysed during this study are included in this published article or in the accompanying Supplementary Information file.
